# Rapid determination of sodium pentachlorophenate in bamboo and wooden cutting boards via ultrasonic-assisted liquid-liquid extraction coupled with ultra-performance liquid chromatography-high resolution mass spectrometry

**DOI:** 10.1371/journal.pone.0326129

**Published:** 2025-06-16

**Authors:** Ye Wang, Mingyou Hu, Fang Wang, Juan Yu, Guojian Shao

**Affiliations:** 1 Huzhou Center for Disease Control and Prevention, Key Laboratory of Emergency Detection for Public Health of Huzhou, Huzhou, China; University of the Witwatersrand Johannesburg, SOUTH AFRICA

## Abstract

Sodium pentachlorophenate (PCP-Na) is a toxic preservative used in wood products, posing potential health risks through food contact materials. A rapid analytical method combining ultrasonic-assisted liquid-liquid extraction with ultra-performance liquid chromatography-high resolution mass spectrometry (UA-LLE-UPLC-HRMS) was developed for the determination of PCP-Na residues in bamboo and wooden cutting boards. Sample pretreatment involved ultrasonic extraction using methanol/water (50:50 v/v, 2.0% ammonia), followed by liquid-liquid purification with n-hexane/ethyl acetate (60:40 v/v). After solvent evaporation under nitrogen, the residue was reconstituted in the initial mobile phase. Chromatographic separation was achieved on an Acquity UPLC BEH C18 column (2.1 mm × 100 mm, 1.7 µm) using a gradient elution of methanol and 0.01% ammoniated aqueous solution. Detection was performed in negative electrospray ionization (ESI^-^) mode with targeted single ion monitoring (Targeted-SIM) scanning, utilizing pentachlorophenol-^13^C_6_ (PCP-^13^C_6_) as an isotopically labeled internal standard. The method exhibited excellent linearity across a concentration range of 1.0–500.0 μg/L (R^2^ ≥ 0.999), with a limit of detection (LOD) of 0.5 μg/kg and a limit of quantification (LOQ) of 1.5 μg/kg. Validation studies at three spiking levels (20.0, 200.0, and 400.0 μg/kg) demonstrated satisfactory recoveries of 97.2%–99.7% and precision with relative standard deviations (RSDs) of 0.8%–1.7% (n = 6). The total chromatographic runtime was optimized to 6 minutes. Application of this method to Seventy-five commercial cutting boards revealed PCP-Na residues in five samples, with concentrations ranging from 1.3 to 416 mg/kg. This approach features streamlined sample preparation, high sensitivity, robust accuracy, and rapid analysis, making it particularly suitable for routine monitoring of PCP-Na residues in bamboo and wooden food contact materials.

## Introduction

Cutting boards serve as primary contact surfaces for diverse food ingredients, making their material safety a critical determinant of public health outcomes. In China, bamboo and wooden cutting boards remain prevalent in household kitchens due to cultural preferences and longstanding usage traditions. Conventionally, these boards are expected to comprise untreated natural materials without synthetic additives. However, their porous organic structure creates inherent challenges: prolonged use with inadequate sanitation promotes microbial proliferation and organic residue accumulation, compromising both hygiene and durability. To address these limitations, certain manufacturers have resorted to incorporating industrial preservatives during production, notably sodium pentachlorophenate (PCP-Na), to inhibit microbial degradation. While effective for material preservation, residual amounts of such biocides on food contact surfaces pose significant consumer health risks. This practice has raised growing concerns amid increasing global scrutiny of food safety, particularly regarding chemical migration from food-contact materials. Recent studies and regulatory reports [1,2] highlight escalating incidents of contamination linked to non-compliant additives in kitchenware, underscoring the urgent need for robust analytical methods to monitor and regulate these hazardous residues.

PCP-Na, a multifunctional organochlorine compound, is extensively utilized as a wood preservative, insecticide, and antibacterial agent in industrial and agricultural applications [3,4]. However, mounting evidence highlights its significant health and environmental risks. PCP-Na exhibits carcinogenic, teratogenic, and genotoxic properties, demonstrating potential to induce chromosomal aberrations, gene mutations, and chronic toxicity through bioaccumulation in biological systems [5–7]. The International Agency for Research on Cancer (IARC) classifies PCP-Na as a Group 2B carcinogen, with particular concern arising from its acidic conversion to pentachlorophenol (PCP), a definitive Group 1 carcinogen [8]. Owing to its environmental persistence and bioaccumulation potential, PCP-Na and its derivatives have become ubiquitous contaminants, detected in aquatic systems, soil matrices, biota, and agricultural products [9–12]. Human exposure occurs primarily via inhalation and ingestion, manifesting acute symptoms including cephalalgia, nausea, and vomiting [13]. Regulatory responses reflect these risks: China prohibits PCP-Na in food animal production and mandates nondetection in animal-derived foods [14], while the U.S. EPA designates it as a priority pollutant [15]. The U.S. EPA does not have a separate drinking water standard for PCP-Na, but lists its parent compound, PCP, as a regulated substance with a maximum contaminant level (MCL) of 0.001 mg/L [16]. WHO has not established a separate limit value for PCP-Na, but proposes the following for PCP: Provisional guideline value: 0.009 mg/L [17]. Commission Delegated Regulation (EU) 2021/277 states that the permissible limit for PCP and its salts and esters equal to or below 5 mg/kg (0,0005% by weight) where they are present in substances, mixtures or articles [18]. Several nations further recognize PCP-Na as both a carcinogen and persistent organic pollutant [19]. The use of PCP-Na as a wood preservative for residential use is banned in countries such as the United States, Canada and Japan [20–22]. Notably, elevated PCP-Na residues persist in bamboo and wooden cutting boards, likely stemming from illegal applications of PCP-Na solutions for corrosion inhibition, mold prevention, and color stabilization during manufacturing. China currently lacks established maximum residue limits (MRLs) for PCP-Na in food-contact materials, creating regulatory vulnerabilities in product quality control. This oversight underscores the urgent need to develop accurate, sensitive analytical methodologies for PCP-Na detection in household food-contact surfaces, ensuring alignment with global food safety standards.

Current analytical approaches for PCP-Na detection encompass gas chromatography (GC) [23], gas chromatography-tandem mass spectrometry (GC-MS/MS) [24,25], liquid chromatography (LC) [26], and liquid chromatography-tandem mass spectrometry (LC-MS/MS) [1,27]. Nevertheless, these methods present operational limitations: GC and GC-MS/MS necessitate derivatization procedures that introduce methodological complexity and susceptibility to matrix interference, while LC suffers from insufficient sensitivity for trace-level analysis. Although LC-MS/MS has gained prominence through its derivatization-free operation, enhanced sensitivity, and superior selectivity, it remains constrained by inadequate molecular weight determination accuracy and limited qualitative confirmation capabilities. Emerging as a robust alternative, quadrupole-orbitrap high-resolution mass spectrometry (Q-Orbitrap HRMS) addresses these limitations through exact mass measurement (<3 ppm mass accuracy), enabling definitive compound identification and eliminating false positives. Simultaneously, its quantitative performance rivals that of triple quadrupole systems. Regarding sample preparation, conventional solid-phase extraction (SPE) methods [28,29], despite widespread adoption, suffer from time-intensive protocols and procedural complexity. This underscores the critical demand for streamlined extraction techniques, particularly solvent-based liquid-liquid extraction (LLE), to enhance throughput without compromising analytical reliability.

Existing analytical investigations of PCP-Na have predominantly focused on environmental matrices and food commodities [30–32], while critical intermediate media like food-contact surfaces (particularly cutting boards) remain underexplored. To address this knowledge gap, we developed a novel ultrasonic-assisted liquid-liquid extraction coupled with ultra-performance liquid chromatography-high-resolution mass spectrometry (UA-LLE-UPLC-HRMS) methodology for quantifying PCP-Na residues in bamboo and wooden cutting boards. The study aims to achieve three objectives: 1) optimize the UA-LLE conditions to streamline pre-treatment steps and enhance detection efficiency. 2) develop an isotope dilution UPLC-HRMS method to improve quantification sensitivity and accuracy, followed by comprehensive methodological validation. 3) assess the contamination levels of PCP-Na in bamboo and wooden cutting boards by analyzing samples collected from the Huzhou area. As a practical application, this validated method was implemented to assess PCP-Na contamination levels in commercially available cutting boards from Huzhou City. The systematic evaluation not only reveals current industrial practices but also establishes foundational data for regulating hazardous substance migration in food-contact materials.

## Materials and methods

### Reagents and materials

HPLC-grade acetonitrile (ACN), methanol (MeOH), n-hexane, and ethyl acetate (EA) were sourced from Merck GmbH (Darmstadt, Germany). HPLC-grade formic acid (FA) was obtained from Shanghai Macklin Biochemical Technology Co., Ltd. (Shanghai, China). Guaranteed reagent-grade ammonia aqueous solution (NH3·H2O) was procured from Merck GmbH (Darmstadt, Germany). Ultrapure water (18.2 MΩ·cm resistivity) was generated using a Milli-Q Integral Water Purification System (EMD Millipore, Billerica, MA, USA). Polytetrafluoroethylene (PTFE) syringe filters (13 mm, 0.22 µm) were purchased from ANPEL Laboratory Technologies (Shanghai) Inc. (Shanghai, China).

A certified reference material of PCP-Na (1000 μg/mL in methanol) was acquired from TMRM Co., Ltd. (Jiangsu, China). The isotopically labeled internal standard, pentachlorophenol-^13^C_6_ (PCP-^13^C_6_, 99% chemical purity), was obtained from Cambridge Isotope Laboratories, Inc. (Andover, MA, USA).

### Instruments and equipment

Vanquish UHPLC system coupled with Q Exactive^TM^ Hybrid Quadrupole-Orbitrap mass spectrometer (Thermo Fisher Scientifi, USA); Multi Reax multi-tube vortex mixer (Heidolph, Germany); KQ-800DE ultrasonic processor (Kunshan Ultrasonic Instruments Co., Ltd., China); Allegra 64R refrigerated centrifuge (Beckman Coulter, Inc., USA); TurboVap LV nitrogen concentrator (Biotage, Sweden); GM200 stainless-steel blade grinder (Retsch GmbH, Germany).

### Standard solutions

The certified PCP-Na standard solution (1000 μg/mL in methanol) was metrologically diluted with anhydrous methanol to achieve a 10-fold diluted stock solution (100 μg/mL). The PCP-^13^C_6_ solid reference material was gravimetrically prepared (purity-corrected mass) in methanol to yield a 100 μg/mL internal standard stock solution. All solutions were cryogenically stored at −20 ± 1 °C in amber glass vials to prevent photodegradation and thermal decomposition.

### Sample preparation and pre-treatment

Seventy-five commercially available bamboo and wooden cutting board specimens were systematically collected through stratified sampling across Huzhou’s administrative divisions, including all three counties (Deqing, Changxing, Anji) and two municipal districts (Wuxing, Nanxun). The sampling campaign (2023–2024) encompassed diverse retail channels: local agricultural markets, supermarket chains, and shopping malls, ensuring representative coverage of consumer-accessible products.

**Fig 1:**
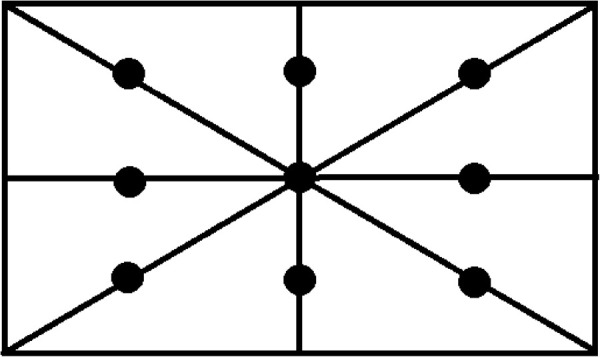
Standardized nine-point grid sampling protocol for rectangular bamboo and wooden cutting boards.

To ensure analytical reliability and minimize cross-contamination risks, the following tiered protocols were rigorously implemented: 1) Tool decontamination: Stainless steel drill bits and collection aluminum foil were pre-cleaned via sequential solvent washing (n-hexane > methanol > ultrapure water, 15 min ultrasonication per cycle) followed by drying. 2) Field blanks: Three procedural blanks (aluminum foil + containers) were processed in parallel with each sampling batch to monitor background contamination. 3) Nine-point grid sampling: Particulate matter was collected from a standardized 3 × 3 grid (Fig. 1) using an electric drill. Drill-derived particulates were immediately deposited on pre-cleaned aluminum foil (pre-washed with n-hexane). 4) Mechanical homogenization with cycle-specific cleaning: Samples underwent three sequential grinding cycles (30 sec at 600 rpm), with inter-cycle chamber decontamination (10 mL methanol rinse + 5 min nitrogen drying) to eliminate particle carryover. 5) Preservation: Homogenized samples were transferred to polyethylene containers, with each sample mass rigorously maintained at ≥20.0 g. All specimens were archived under controlled ambient conditions (25 ± 2°C, RH < 40%) pending analysis.

Accurately weighed homogenized samples (0.5 ± 0.001 g) were transferred to 50 mL polypropylene centrifuge tubes. Subsequently, 50 μL of isotopically labeled internal standard working solution (2000 μg/L PCP-^13^C_6_) and 10 mL of a methano/water (50:50 v/v, 2.0% ammonia) were added. The mixture underwent primary extraction through sequential processing: 1) vortex homogenization (2500 rpm, 60 s); 2) ultrasonic-assisted extraction (20 kHz, 450 W, 10 min); and 3) centrifugation (10,000 rpm, 5 min, 4 °C). A 5.00 mL aliquot of the supernatant was subjected to liquid-liquid extraction in 15 mL PP tubes with 75 μL formic acid and 4 mL n-hexane/ethyl acetate (60:40 v/v). Following secondary vortex mixing (5 min) and centrifugation (10,000 rpm, 5 min), the organic phase was quantitatively transferred to fresh tubes and concentrated to dryness under nitrogen (30 °C, 15 psi) for 45 min. The residue was reconstituted in 1.00 mL of methanol/0.01% ammonia aqueous solution (v/v 50:50) and then filtered through 0.22 μm PTFE syringe filters into certified LC vials for UPLC-HRMS analysis.

### Analysis parameters of instruments

Chromatographic separation was performed on an Acquity UPLC BEH C18 analytical column (2.1 × 100 mm, 1.7 μm particle size;) maintained at 30.0 ± 0.5 °C. The mobile phase consisted of (A) 0.01% ammonia aqueous solution and (B) methanol, delivered at 0.300 mL/min with a 5.00 μL injection volume. A six-step gradient elution program was implemented as detailed in Table 1, achieving complete separation within 6.0 min runtime.

**Table 1:** Gradient elution conditions.

Time (min)	Flow (mL/min)	Phase A/%	Phase B/%
0	0.300	50.0	50.0
1	0.300	50.0	50.0
3	0.300	10.0	90.0
4	0.300	10.0	90.0
5	0.300	50.0	50.0
6	0.300	50.0	50.0

High-resolution mass spectrometry was performed on a Q Exactive^TM^ Hybrid Quadrupole-Orbitrap in negative electrospray ionization (ESI^−^) mode. Optimized parameters included a 3.0 kV spray voltage, sheath gas (N_2_) at 45 Arb, auxiliary gas at 10 Arb, S-lens RF level 55, auxiliary gas heater at 350 °C, and capillary temperature at 320 °C. Data acquisition employed a targeted single ion monitoring (Targeted-SIM) with a 4.0 m/z isolation window, m/z 50–750 scan range, 200 ms ion injection time (IT), automatic gain control (AGC) target of 5 × 10^4^ ions, and 70,000 full width at half maximum (FWHM) resolution at m/z 200.

### Method validation

Validation parameters were then evaluated according to FDA guidelines [33]. To validate the analytical method of PCP-Na in bamboo and wooden cutting boards, blank samples were selected, and the verification parameters such as linearity, accuracy, precision, limits of detection (LOD), and limits of quantification (LOQ) were assessed. Linearity is the assumption that there is a straightline relationship between the input (x) and output (y) variables. It is common practice to check the linearity of a calibration curve by inspection of the correlation coefficient r (R^2^). Meanwhile, Analytical Method Committee suggests using the F-test as a reliable approach to check the linearity of any calibration function [34]. Accuracy is the degree of agreement between the experimental value, obtained by replicate measurements, and the accepted reference value. The accuracy is usually estimated by spiking a blank sample with low, medium or high concentration levels of the substance to be measured and by recovery tests. Precision is defined as the closeness of agreement between quantity values obtained by replicate measurements of a quantity under specified conditions. The relative standard deviation (RSD) of six replicate samples at each spiked level is commonly used to assess analytical method precision. The LOD is commonly defined as the lowest amount of analyte in a sample that can be reliably detected but not necessarily quantitated by a particular analytical method. The LOQ is defined as the lowest concentration or amount of analyte that can be determined with an acceptable level of precision and accuracy. The LOD and LOQ were established through experimental determination based on the signal-to-noise ratio (LOD = 3 S/N and LOQ = 10 S/N, the LOD and LOQ of the method were converted according to the volume (V), the conversion factor (f) and the weighing volume (m)).

### Statistical analysis

All results were presented as the mean of three or six independent experiments. Excel 2019 and OriginPro 2024 were used for chart drawing. The error bars of the figures were generated by the values of the standard deviation.

## Results and discussion

### Optimization of chromatographic conditions

The BEH C18 stationary phase (2.1 × 100 mm column geometry; 1.7-μm particle size) demonstrated superior chromatographic performance, producing excellent peak shape and high S/N ratio. Based on these analytical merits, this UPLC-optimized column configuration was ultimately implemented for compound separation.

**Fig 2:**
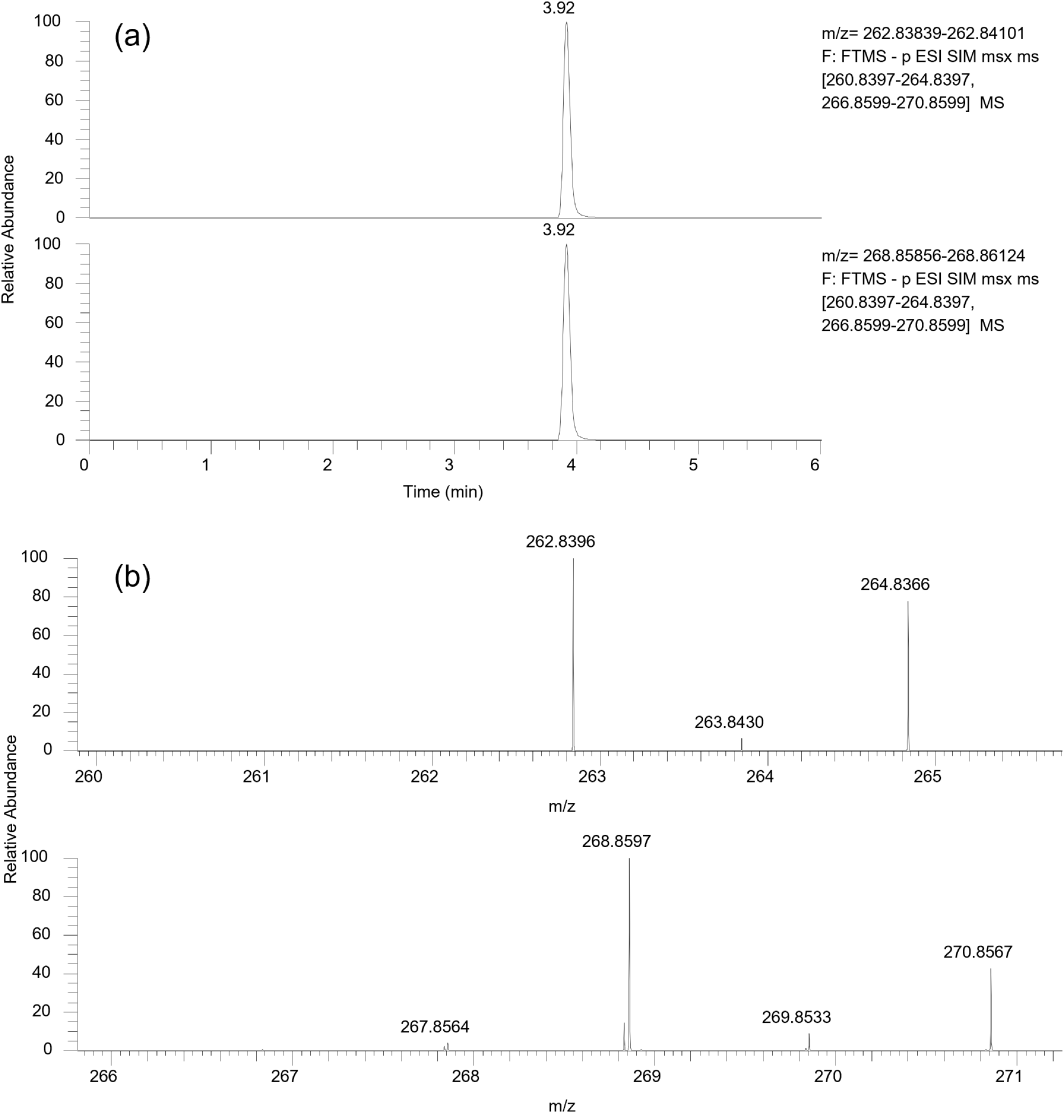
SIM chromatograms (a) and mass spectra (b) of PCP-Na and PCP-^13^C_6_ with a mass concentration of 50 µg/L. PCP-Na and PCP-^13^C_6_ retention time: 3.92 min; m/z: 262.8396 (−0.4 ppm) and 268.8597 (−0.8 ppm).

The study systematically evaluated four mobile phase compositions—methanol/water, methanol/0.01% ammonia aqueous solution, acetonitrile/water, and acetonitrile/0.01% ammonia aqueous solution—for their chromatographic and mass spectrometric performance with PCP-Na and PCP-^13^C_6_. Results indicated that methanol-based mobile phases provided optimal elution strength. Specifically, the methanol/0.01% ammonia aqueous solution combination yielded superior performance, demonstrating enhanced peak symmetry, higher ionization efficiency, and narrower peak widths compared to non-ammoniated systems. This improvement is attributed to ammonia’s role in promoting deprotonation of the phenolic hydroxyl groups, which facilitates ionization in ESI^-^ mode. SIM chromatograms of PCP-Na and PCP-^13^C_6_ with a mass concentration of 50 µg/L under the optimized conditions are shown in Fig 2.

### Optimization of mass spectrum conditions

The molecular architecture of pentachlorophenol incorporates five chlorine atoms, whose natural isotopic distribution (^35^Cl and ^37^Cl) generates characteristic isotopic clusters with distinct exact mass-to-charge ratios. Leveraging the Orbitrap detector’s high mass resolution capability, monoisotopic ^35^Cl selection was implemented during analysis. Targeted-SIM scans in ESI^-^ mode specifically captured the desodiated [M-Na]^-^ ions for PCP-Na and deprotonated [M-H]^-^ ions for PCP-^13^C_6_ in standard solutions. Mass accuracy errors between observed and theoretical exact masses remained below 3 ppm (Fig 2), with absolute mass deviations under 0.5 mDa. Mass spectral information (Table 2) is provided for further details.

**Table 2:** Mass spectral information of PCP-Na and PCP-^13^C_6_.

Compound	Quasi-molecular Ion	Theoretical Exact Mass (m/z)	Experimental Exact Mass (m/z)	Deviation (ppm)
PCP-Na	[M-Na]^-^: C_6_Cl_5_O^-^	262.8397	262.8396	−0.4
PCP-^13^C_6_	[M-H]^-^: ^13^C_6_Cl_5_O^-^	268.8599	268.8597	−0.8

### Optimization of pre-treatment conditions

#### Optimization of extraction solvents.

**Fig 3:**
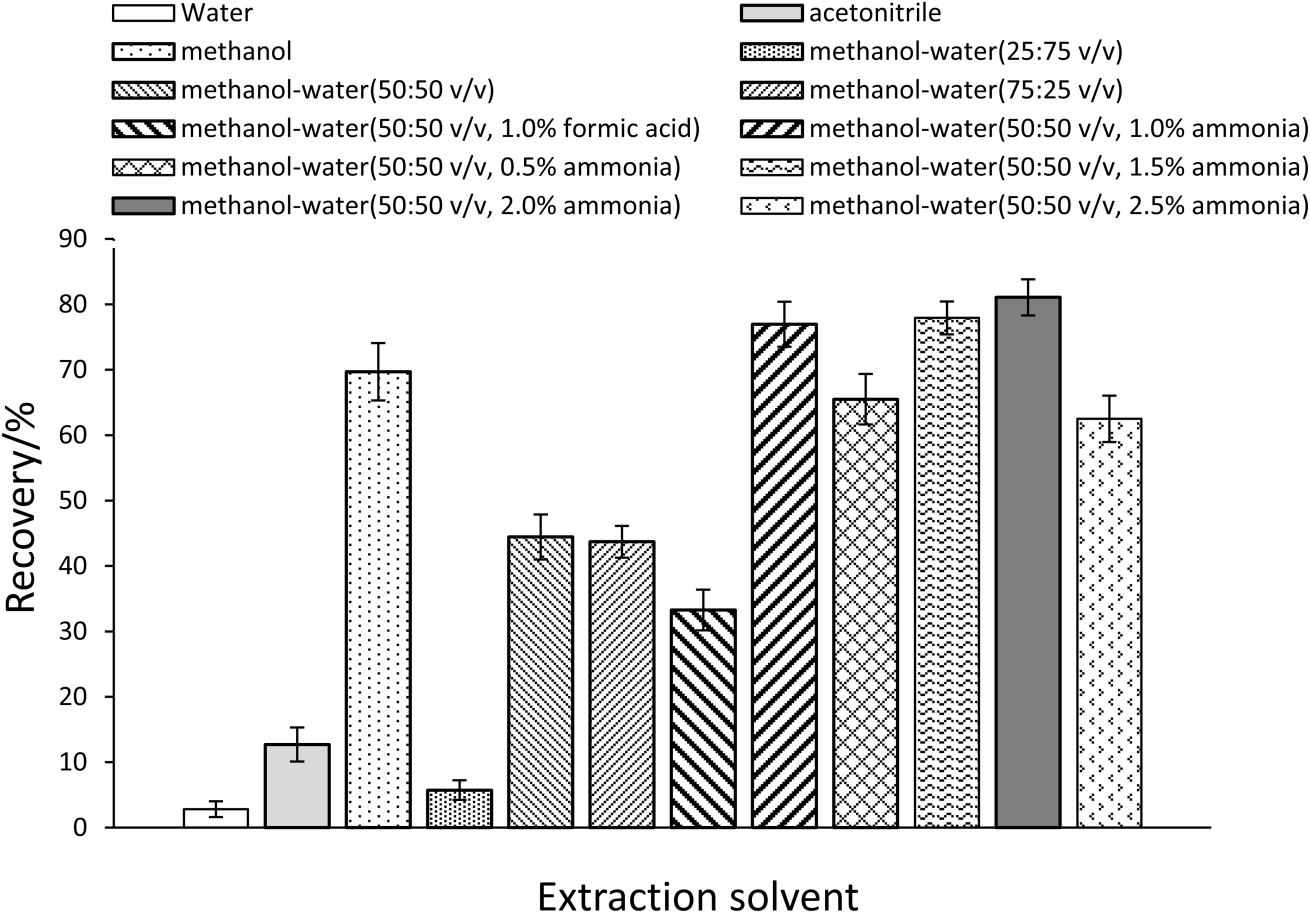
The recovery of PCP-Na with different extraction solvents (n ** = 3).** The recovery was calculated as mean values. The optimal recovery (%) was 81.1 ± 2.8.

A systematic screening of extraction solvents was conducted to optimize PCP-Na recovery at 50 μg/L spiking levels. Evaluated systems included: (1) neat solvents (water, acetonitrile, methanol), (2) methanol/water mixtures (25:75, 50:50, 75:25 v/v), and (3) acid/base-modified methanol/water (50:50 v/v, 1.0% formic acid or 1.0% ammonia). Initial results demonstrated that methanol/water (50:50 v/v, 1.0% ammonia) achieved 77.0% recovery, outperforming other solvents by 11.1–74.1% (Fig 3). Subsequent methodological refinement through ammonia concentration optimization (0.5–2.5% v/v) revealed peak recovery (81.1%) at 2.0% ammonia, attributable to enhanced phenolic group deprotonation and improved phase partitioning efficiency. This optimized solvent system (methanol/water 50:50 v/v, 2.0% ammonia) was consequently selected for all subsequent analyses.

#### Optimization of ultrasonic extraction time.

**Fig 4:**
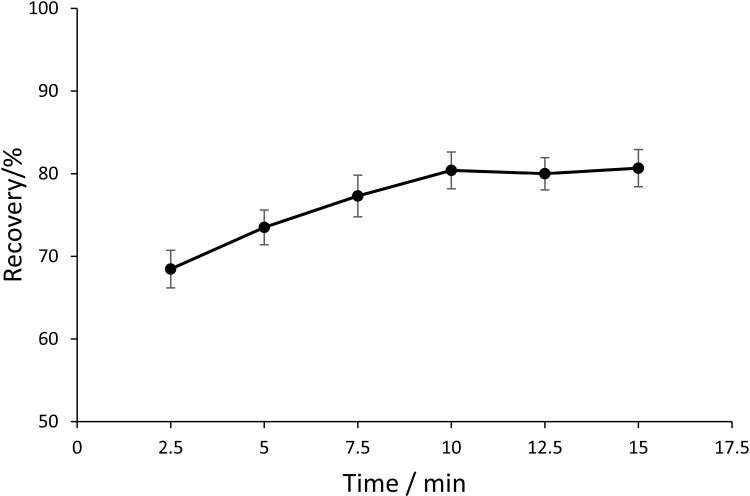
The recovery of PCP-Na with different ultrasonic extraction times (n ** = 3).** The recovery was calculated as mean values. The optimal recovery (%) was 80.4 ± 2.2.

The influence of ultrasonic extraction duration on PCP-Na recovery was systematically investigated across a time gradient (2.5–15 min). As illustrated in Fig 4, analyte recovery exhibited a time-dependent enhancement, increasing from 68.5% at 2.5 min to 80.4% at 10 min. Beyond 10 min, recovery plateaued (80.7% at 15 min), indicating that 10 min represents the optimal balance between extraction efficiency and operational practicality. Consequently, 10 min was established as the standardized extraction duration.

### Optimization of the formic acid volume addition and liquid-liquid extraction solvent composition

**Fig 5:**
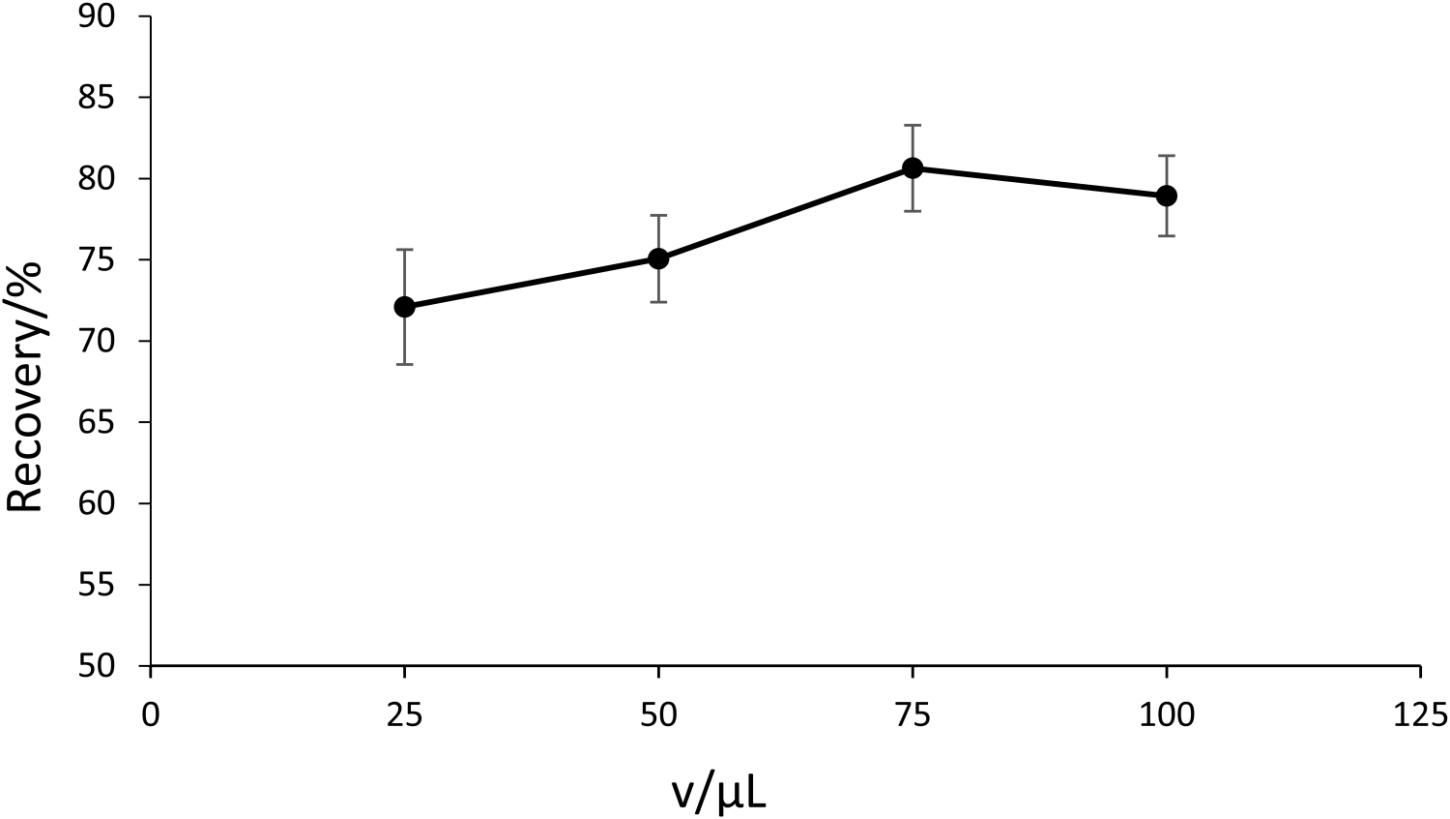
The recovery of PCP-Na with different volumes of formic acid added (n ** = 3).** The recovery was calculated as mean values. The optimal recovery (%) was 80.6 ± 2.7.

**Fig 6:**
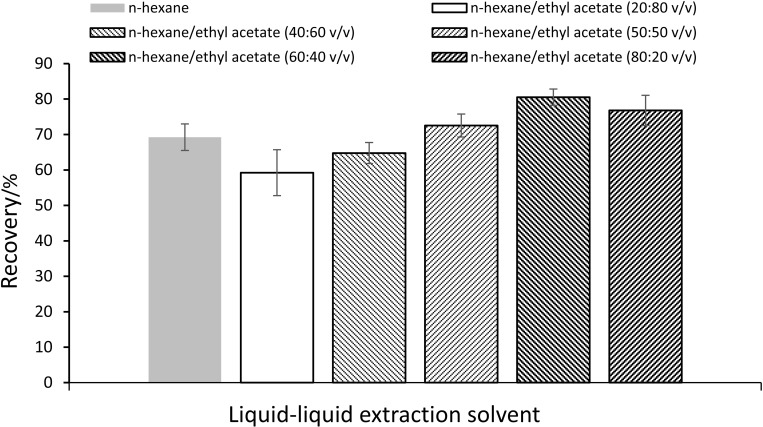
The recovery of PCP-Na with different liquid-liquid extraction solvent composition (n ** = 3).** The recovery was calculated as mean values. The optimal recovery (%) was 80.5 ± 2.3.

A two-stage optimization protocol was implemented to enhance PCP-Na recovery during liquid-liquid extraction (LLE). Initial acidification studies evaluated the impact of formic acid volume (25–100 μL) addition prior to LLE on PCP-Na recovery. As shown in Fig 5, recovery increased proportionally with acid volume, peaking at 75 μL (80.6% recovery), beyond which a 1.7% decline occurred at 100 μL. Subsequent liquid-liquid extraction solvent optimization compared six n-hexane/ethyl acetate ratios (20:80–100:0 v/v). The 60:40 v/v system demonstrated optimal performance (80.5% recovery). This ratio provided 4.8–21.3% higher recovery than other combinations, as shown in Fig 6. The results showed that n-hexane/ethyl acetate (60:40 v/v) was selected as the standardized LLE protocol, followed by nitrogen blow-down at room temperature.

### Method validation

#### Linearity, LOD, and LOQ.

To prepare calibration solutions, appropriate amounts of PCP-Na external standard and PCP-^13^C_6_ internal standard were diluted in a methanol-0.01% ammonia solution (50:50 v/v). The internal standard concentration was set at 50.0 µg/L, while the PCP-Na concentrations were set at 1.0, 5.0, 10.0, 20.0, 50.0, 100.0, 200.0 and 500.0 µg/L. UPLC-HRMS in Targeted-SIM mode, with the quasi-molecular ions [M-Na]^-^ at m/z 262.8397 for PCP-Na and [M-H]^-^ at m/z 268.8599 for the isotopically labeled internal standard PCP-^13^C_6_. Calibration curves were constructed by plotting the peak area ratio (Y = analyte/internal standard) against the analyte concentration (X, µg/L), employing 1/X weighting to account for heteroscedasticity. The results showed that PCP-Na exhibited a good linear relationship within the range of 1.0–500.0 µg/L, with a regression equation of Y = 0.00835845X + 9.369e-5 and a linear determination coefficient (R^2^) of 0.9998. The linearity of the standard curve was verified by an F-test (Table 3). Blank bamboo and wooden cutting boards were spiked with low concentrations for detection. Method validation studies revealed a LOD of 0.5 μg/kg and a LOQ of 1.5 μg/kg, respectively.

**Table 3:** F-test for linearity.

Comments			X	Y			$\overset{―}{Y}$		
The amount of analyte (μg/L), the chromatographic analyte/internal standard peak area ratio and its average are designated as X, Y and $\overset{―}{Y}$ respectively.			1	0.0084	0.0091	0.0082	0.0086		
			5	0.0418	0.0420	0.0415	0.0418		
			10	0.0833	0.0842	0.0836	0.0837		
			20	0.1669	0.1654	0.1676	0.1666		
			50	0.4199	0.4170	0.4184	0.4184		
			100	0.8321	0.8365	0.8383	0.8356		
			200	1.6840	1.6800	1.6710	1.6783		
The calibration curve was obtained by plotting y vs x. Proposed linear model by using the reported data. Reported squared correlation coefficient (R^2^).			500	4.1848	4.1747	4.1798	4.1798		
			$\hat{Y}=0.00835845X+9.369e-5$						
				R^2^ = 0.9998					
							
**X**	${\left(Y-\hat{Y}\right)}^{2}$			${\left(Y-\overset{―}{Y}\right)}^{2}$			${\left(\overset{―}{Y}-\hat{Y}\right)}^{2}$		
1	2.72e-9	4.20e-7	6.36e-8	4.00e-8	2.50e-7	1.60e-7	2.19e-8	2.19e-8	2.19e-8
5	7.39e-9	1.30e-8	1.49e-7	0.00	4.00e-8	9.00e-8	7.39e-9	7.39e-9	7.39e-9
10	1.43e-7	2.72e-7	6.11e-9	1.60e-7	2.50e-7	1.00e-8	4.76e-10	4.76e-10	4.76e-10
20	1.32e-7	3.47e-6	1.14e-7	9.00e-8	1.44e-6	1.00e-6	4.39e-7	4.39e-7	4.39e-7
50	3.55e-6	1.03e-6	1.47e-7	2.25e-6	1.96e-6	0.00	1.47e-7	1.47e-7	1.47e-7
100	1.47e-5	3.15e-7	5.58e-6	1.23e-5	8.10e-7	7.29e-6	1.15e-7	1.15e-7	1.15e-7
200	1.49e-4	6.75e-5	6.14e-7	3.25e-5	2.89e-6	5.33e-5	4.25e-5	4.25e-5	4.25e-5
500	3.00e-5	2.13e-5	2.32e-7	2.50e-5	2.60e-5	0.00	2.32e-7	2.32e-7	2.32e-7
Residual error sum squares (Eq. 1) Pure error sum squares (Eq. 2) Lack-of-fit error sum squares (Eq. 3)	$\begin{array}{c} {SS}_{r}=\sum_{i=1}^{I}\sum_{j=1}^{j_{i}}{\left(Y_{ij}-\hat{Y_{i}}\right)}^{2} \end{array}$ (Eq. 1)			$\begin{array}{c} {SS}_{\varepsilon }=\sum_{i=1}^{I}\sum_{j=1}^{j_{i}}{\left(Y_{ij}-\overset{―}{Y_{i}}\right)}^{2} \end{array}$ (Eq. 2)			$\begin{array}{c} {SS}_{lof}=\sum_{i=1}^{I}\sum_{j=1}^{j_{i}}{\left(\overset{―}{Y_{i}}-\hat{Y_{i}}\right)}^{2} \end{array}$ (Eq. 3)		
	${SS}_{r}=2.99e-4$			${SS}_{\varepsilon }=1.68e-4$			${SS}_{lof}=1.30e-4$		
Degrees of freedom (DF)	${DF}_{r}=IJ-2=24-2=22$			${DF}_{\varepsilon }=IJ-I=24-8=16$			${DF}_{lof}=I-2=8-2=6$		
Associated variances $\sigma^{2}=SS/DF$	$\sigma_{r}^{2}=1.36e-5$			$\sigma_{\varepsilon }^{2}=1.05e-5$			$\sigma_{lof}^{2}=2.17e-5$		
Fisher ratio ($F=\sigma_{lof}^{2}/\sigma_{lof}^{2}$) calculated (if F_calculated_ < F_tabulated_ then Linear)	2.067 (calculated) < 2.741 (tabulated at the 95% with 6 and 16 degrees of freedom)								
Conclusions	R^2^ = 0.9998, while F_calculated_ < F_tabulated_, indicating that the curve is linear								

#### Accuracy and precision.

To evaluate method accuracy and precision, matrix recovery studies were conducted using blank bamboo and wooden cutting boards fortified with PCP-Na at three concentration levels (20.0, 200.0, and 400.0 μg/kg). Six independent replicates per level were prepared by spiking with PCP-Na standard and PCP-^13^C_6_ internal standard solutions, followed by sample pretreatment and UPLC-HRMS analysis. As summarized in Table 4, the method demonstrated exceptional accuracy with mean recoveries of 97.2%–99.7% across all tested concentrations. Precision was confirmed by low RSDs ranging from 0.8% to 1.7% (n = 6). These results collectively validate the effectiveness of the isotope dilution strategy using ^13^C_6_-labeled internal standard. The observed recovery consistency across distinct concentration levels indicates minimal matrix interference, attributable to the structural analog compensating for extraction efficiency variations and ionization suppression/enhancement effects during MS detection. Furthermore, the sub-2% RSD values highlight the method’s robustness against operational variability in complex sample processing workflows.

**Table 4:** The recovery and RSD of PCP-Na in bamboo and wooden cutting boards at different spiked levels (n = 6).

Compound	Spiked Level (μg/kg)	Average Recovery (%)	RSD (%)
PCP-Na	20.0	99.7	1.7
	200.0	97.5	0.8
	400.0	97.2	0.9

### Determination comparison to other methods

Table 5 summarizes a comparative analysis of existing methods for determining pentachlorophenol and its sodium salt in wooden products. The LODs of the existing methods were in the range of 0.2–3.0 μg/kg. The sensitivity of this method was slightly lower than that of previously reported methods [1,25]. This reduced sensitivity is associated with the sample amount used during pre-treatment. The simplified pre-treatment workflow based on UA-LLE greatly simplified the sample purification process and gave the method better precision (0.8% to 1.7%). Notably, the combination of HRMS calibrated with isotopically labeled internal standards effectively mitigates matrix effects and improves the accuracy and reliability of quantification, resulting in better average recoveries (97.2%−99.7%) for this method, compared to methods based on external standards.

**Table 5:** Comparison of proposed method respect to references published by others.

Matrix	Equipment	Sample preparation	Quantification	LOD (μg/kg)	LOQ (μg/kg)	Recovery (%)	RSD(%)	Reference
Surface of wooden chopping board	GC-MS/MS	Purification by SLC SPE column and acetic anhydride and pyridine derivatization	Internal standard curve	3.0	10.0	86.0% ~ 96.0%	2.% ~ 4.2%	[24]
Wooden chopping boards and wooden chopsticks	GC-MS/MS	Vortex-assisted pre-column derivatization	Internal standard curve	0.2	0.7	90.0% ~ 103.6%	1.5% ~ 3.6%	[25]
Cutting boards	UPLC-MS/MS	Extracted using solvent and purification by automated SPE system.	Matrix-matching internal standard curve	0.4	1.0	71.75% ~ 96.50%	5.19% ~ 16.66%	[1]
Wooden chopsticks	UPLC-HRMS	Ultrasonically extracted and purification by SLC SPE column	External standard curve	2.0	6.0	80.7% ~ 95.3%	6.6%	[35]
Bamboo and wooden cutting boards	UPLC-HRMS	Ultrasonic-assisted liquid-liquid extraction	Internal standard curve	0.5	1.5	97.2% ~ 99.7%	0.8% ~ 1.7%	This study

### Actual sample determination

The validated method was implemented to assess PCP-Na contamination in seventy-five commercially available bamboo and wooden cutting boards collected across five administrative regions (Wuxing District, Nanxun District, Changxing County, Deqing County, and Anji County) of Huzhou City between 2023 and 2024. The sampling strategy comprised two phases: 25 samples (5 per region) in 2023 and 50 samples (10 per region) in 2024. Notably, contamination hotspots were identified in Nanxun District and Anji County through longitudinal monitoring. In the 2023 cohort, two samples were quantified above the LOD: one from Nanxun District (1.3 mg/kg) and one from Anji County (183 mg/kg). The 2024 survey revealed that three samples exceeded the LOD, including two from Nanxun District (13.4 mg/kg and 74.6 mg/kg) and one from Anji County (416 mg/kg). These findings underscore the critical need for enhanced regulatory oversight in food-contact material production chains.

## Conclusions

This study successfully developed a rapid and robust analytical method for monitoring PCP-Na residues in bamboo and wooden food-contact materials. By integrating ultrasound-assisted liquid-liquid extraction (UA-LLE) with UPLC-HRMS detection, this method achieves high sensitivity (LOD: 0.5 μg/kg) and precision (RSD < 2%), significantly simplifying sample preparation.The optimized workflow minimizes solvent consumption and achieves chromatographic separation within 6 minutes, demonstrating high efficiency for high-throughput screening.Analysis of 75 commercially available bamboo and wooden cutting boards from Huzhou City revealed that five samples contained detectable PCP-Na, with concentrations ranging from 1.3 mg/kg to 416 mg/kg. According to Regulation (EU) 2021/277, the permissible limit is 5 mg/kg. Among the detected samples, four exceeded the 5 mg/kg limit by a significant margin, highlighting potential risks associated with PCP-Na residues in bamboo and wooden cutting boards and the need for regulatory attention. This study provides empirical evidence advocating for stricter regulations on chlorophenol-based preservatives in household food-contact materials.

## Supporting information

S1 Data Minimal data set. (XLSX)
